# MAPK/ERK pathway is necessary to the reconsolidation of fear memory in male and female rats

**DOI:** 10.1007/s00213-025-07002-7

**Published:** 2026-01-22

**Authors:** Jaqueline Rauber Ludwig, Thais Fernanda Dalferth, Giovana Vitória Rodrigues Fujita, Cristiane Regina Guerino Furini

**Affiliations:** 1 Laboratory of Cognition and Memory Neurobiology, Brain Institute, Pontifical Catholic University of Rio Grande do Sul (PUCRS), Av. Ipiranga, 6690 – Bldg. 63, 3rd floor, Porto Alegre, 90610-000 RS Brazil; 2 Institute of Geriatrics and Gerontology, Pontifical Catholic University of Rio Grande do Sul (PUCRS), Av. Ipiranga, 6681 – Bldg. 40, 8th floor, Porto Alegre, 90610-000 RS Brazil; 3 Brain Institute - Pontifical Catholic University of Rio Grande do Sul (PUCRS), Av. Ipiranga, 6690 – Bldg. 63, 3rd floor, Porto Alegre, RS 90610-000 Brazil

**Keywords:** Memory, Reconsolidation, Male and female rats, MAPK/ERK

## Abstract

**Rationale:**

Fear memories are essential for survival, but their inappropriate expression can contribute to anxiety disorders, phobias, and post-traumatic stress disorders that can present differently between the sexes. Memory reconsolidation is a process through which previously stored memories can be modified upon reactivation. The mitogen-activated protein kinase/extracellular signal-regulated kinase (MAPK/ERK) signaling pathway regulates neuronal gene transcription and is involved in synaptic plasticity and memory formation. Evidence suggests possible sex differences in the neurobiological mechanisms underlying fear responses and memory processes. However, its involvement in the reconsolidation of contextual fear conditioning (CFC) memory in male and female rats remains unclear.

**Objective:**

To investigate the involvement of the MAPK/ERK pathway in the CA1 region of the dorsal hippocampus during CFC memory reconsolidation in male and female Wistar rats.

**Methods:**

The role of the MAPK/ERK pathway was evaluated by infusing the selective inhibitor U0126 (0.04 µg/side) into the hippocampus at different time points after the reactivation session.

**Results:**

The results demonstrated that, in both male and female rats, the inhibition of the MAPK/ERK pathway in the CA1 region impaired the CFC memory reconsolidation when administered immediately and 3 h after the reactivation session. However, this effect was not observed when administered 6 h later.

**Conclusions:**

These findings indicate the involvement of the MAPK/ERK signaling pathway in the CA1 region of the hippocampus in the reconsolidation of contextual fear conditioning memory in male and female rats and provide new insights into the molecular mechanisms underlying memory reconsolidation, highlighting a common molecular mechanism across sexes.

## Introduction

Memory is an essential cognitive process that enables the acquisition, storage, and retrieval of information throughout time (Izquierdo et al. 2006a). It is not a static phenomenon but rather a dynamic one, influenced by environmental factors which can affect the way information is encoded, stored, and later reactivated (McGaugh 2000; Paller and Voss 2004; Meyer and Louilot 2014; Ferreira et al. 2019; Dalferth et al. 2024).

Acquisition is the initial phase of this process, during which new information is encoded in the brain. This occurs immediately after exposure to a stimulus or event that can lead to learning and, subsequently, to a stable memory (McGaugh 2000). This process is called memory consolidation, which involves various intracellular processes, such as the activation of phosphorylation cascades and protein synthesis, which establish pathways for accessing stored information (McGaugh 1966; Dudai 2004; Squire et al. 2015). Subsequently, we can access these pathways for memory retrieval, where the information is brought back into consciousness (Furini et al. 2013; Fernandes et al. 2022). In this context, during retrieval, memories can be reactivated and temporarily become labile, making them susceptible to modifications, a process known as reconsolidation (Sara 2000; Lee et al. 2004; Dudai and Eisenberg 2004; Izquierdo et al. 2006b; Nader and Hardt 2009; Alberini 2011; Furini et al. 2020). This return to a labile state has practical relevance for the study and treatment of maladaptive emotional memories, such as those involved in panic disorder, generalized anxiety, and post-traumatic stress disorder (Maren 2011; Izquierdo et al. 2016; Kida 2022; Giachero et al. 2024). The context-dependent contextual fear conditioning paradigm is an experimental model that generates memories resembling those observed in these pathologies and allows the investigation of interventions targeting memory reconsolidation (Briscione et al. 2014). According to previous studies, the CA1 region of the dorsal hippocampus is involved in fear memory consolidation and reconsolidation (Izquierdo and Medina 1993; Shimizu et al. 2000; Debiec et al. 2002; Lee 2010).

The mitogen-activated protein kinase/extracellular signal-regulated kinase (MAPK/ERK) pathway is part of an intracellular phosphorylation cascade involved in synaptic transmission, neuronal gene expression, and protein synthesis in response to extracellular stimuli. Its classical activation occurs through tyrosine kinase receptors (Trk), which trigger a phosphorylation sequence involving the G protein Ras, mitogen-activated protein kinase kinase kinase (MAPKKK, Raf-1), and the protein kinase kinase (MAPKK, MEK), ultimately leading to activation of the enzyme MAPK (ERK) (Marshall 1995; Shaywitz and Greenberg 1999; Jiang and Hunter 1999; Orton et al. 2005; Zarich et al. 2006; Eblen 2018). Once activated, ERK can translocate to the nucleus and stimulate transcription factors such as cAMP response element-binding protein (CREB), thereby promoting protein synthesis required for long-term synaptic plasticity. This pathway also regulates several cellular processes, including those related to retrieval-induced memory (Ojea Ramos et al. 2022).

Studies provide evidence that the MAPK/ERK pathway contributes to learning, memory consolidation, and reconsolidation (Bozon et al. 2003; Kelly et al. 2003). This association can be attributed to the dependence of these processes on protein synthesis and the role of MAPK/ERK in transducing extracellular signals to activate protein synthesis (Davis and Squire 1984; Nader et al. 2000). The importance of this pathway has been demonstrated in both the consolidation and reconsolidation across different types of memory (Blum et al. 1999; Bozon et al. 2003; Kelly et al. 2003; Atkins et al. 1998; Schafe et al. 2000; Kida et al. 2002). Regarding the reconsolidation of fear memories, a study demonstrated a central role of the MAPK/ERK pathway in auditory fear memory conditioning through the intraperitoneal infusion of SL327 (MEK inhibitor) after memory reactivation which compromised the reconsolidation of fear memories in wild-type mice, as well as in ERK1 knockout mice, suggesting that ERK2 is the central component in this process (Cestari et al. 2006). In the same paradigm, intraperitoneal administration of mirdametinib combined with prediction error compromised weak fear memory and reduced ERK phosphorylation in the lateral amygdala. In contrast, for strong fear memory, the interruption required the combined treatment of mirdametinib, D-cycloserine, and prediction error (Raut et al. 2024). In a fear conditioning paradigm with two distinct auditory memories, intra-lateral amygdala (intra-AL) infusion of U0126 before retrieval selectively affected the reconsolidation of the reactivated memory and reduced the field potentials evoked in LA in response to the corresponding conditioned stimulus (Doyère et al. 2007). Furthermore, infusion of U0126, a selective MAPK/ERK inhibitor, into the basolateral amygdala of rats demonstrated the role of this pathway in auditory fear memory reconsolidation, showing that its inactivation in this structure impaired long-term memory (Duvarci et al. 2005).

Additionally, preclinical studies investigating aspects of memory are predominantly conducted in male experimental models. However, the inclusion of female models in biological research has become essential due to observed sex differences, such as findings demonstrating differences in the prevalence of neuropsychiatric conditions (Maeng and Milad 2015). Considering sex as a biological variable in experimental studies is crucial to avoid limitations in the translational applicability of preclinical biomedical research. In response to this, new guidelines and recent scientific findings emphasize the importance of incorporating female models in experimental investigations (Clayton 2016). In experimental studies that included both male and female rats, research has demonstrated similar effects of cannabidiol (CBD) on fear memory reconsolidation across sexes (Franzen et al. 2022). However, other findings suggest sex-specific differences in training efficiency and memory acquisition in the Morris water maze task (Flint et al. 2007).

Therefore, these findings highlight the need to expand the understanding of potential physiological and behavioral differences between females and males, including those related to memory. Considering that the role of the MAPK/ERK signaling pathway in the reconsolidation of contextual fear conditioning memory in males and females remains unclear, this study investigated the involvement of the MAPK/ERK signaling pathway in CFC memory reconsolidation, in both male and female rats, in the CA1 region of the dorsal hippocampus, through a pharmacological approach.

## Methodology

### Subjects

Male and female Wistar rats (CrlCembe: WI; 300–330 g) from Centro de Modelos Biológicos e Experimentais (CeMBE) of the Pontifical Catholic University of Rio Grande do Sul, Porto Alegre – Brazil, were housed four per cage. The animals had free access to food and water, and the animal room was maintained at 22–24 °C with a 12-h light–dark cycle (lights on at 7:00 a.m.). All experimental procedures were approved by Animal Committee on Ethics for the Care and Use of Laboratory Animals of the Pontifical Catholic University of Rio Grande do Sul, Brazil in compliance with National Institutes of Health Guidelines for the care and use of laboratory animals.

### Surgery

The animals were submitted to a stereotaxic surgery for the bilateral implantation of stainless steel 22-gauge guide cannulae aimed 1.0 mm above of the CA1 region of the dorsal hippocampus (anterior − 4.2 mm; lateral ± 3.0 mm; ventral − 1.8 mm) (Paxinos and Watson 2014). For the procedures, the animals were anesthetized by intraperitoneal (i.p.) infusion of 75 mg/kg of ketamine plus 10 mg/kg of xylazine. The guide cannulae were fixed to the skull with dental acrylic. Meloxicam was administered at a dose of 1 mg/kg once a day for two days after surgery for pain treatment. After the procedure, animals were allowed 7 days to recover and were handled once daily for 3 consecutive days before behavioral experiments.

### Pharmacological interventions

The drug used was U0126 (1,4-diamino-2,3-dicyano-1,4-bis[2-aminophenylthio]butadiene, 0.04 µg/side) (Igaz et al. 2002), a selective inhibitor of MAPK/ERK (Duncia et al. 1998). The animals in the control group received equal volumes of DMSO 1% in sterile saline solution (0.9%), the same vehicle (Veh) used for dissolving the drug.

The volume of drug or Veh infused was 1.0 µl/side, in the CA1 region of the dorsal hippocampus. For the infusions, Hamilton^®^ microsyringes were used, attached to a polyethylene tube with an infusion needle (0.05 mm in diameter). The needles extended 1.0 mm beyond the tip of the guide cannula fixed to the skull. The infusions were performed at a rate of 0.5 µl/30 s. At the end of the infusion, the needles were kept inside the guide cannula for an additional 60 s to prevent fluid reflux and ensure proper drug diffusion.

### Reconsolidation of contextual of fear conditioning

Contextual fear conditioning (CFC) was performed in a conditioning chamber with aluminum walls (0.35 × 0.35 × 0.35 m) and a clear front lid. The floor of the chamber was made of parallel stainless-steel grid bars and connected to a shock source for the delivery of footshock. The conditioning chamber was placed inside a sound-attenuating box with a ventilating fan. To avoid the interference of smells, the chamber was cleaned with 70% ethanol before and after each use.

For the training session (day 1), animals were placed in the conditioning chamber, and after 2 min, three electrical footshocks (0.7 mA/2 s) were delivered at a 30 s interval. After the last stimulus, the animals remained for 30 additional seconds in the chamber before returning to their home cages. After 24 h (day 2), animals in the reactivated group were placed in the same conditioning chamber for a 3-minute reactivation session of CFC in the absence of footshock and then received infusions in the CA1 region of the hippocampus. The animals in the non-reactivated group did not undergo the reactivation session, receiving only the infusions in the CA1 region and then returning to their home cages. After 24 h (day 3), the animals were placed back in the same apparatus for a 3-minute retention test, again without footshock. Animals were randomly assigned to control (Veh) or drug groups.

During the experimental sessions, the time animals spent immobile in the apparatus (without any visible movement except for breathing) was recorded by an experienced researcher who was unaware of the animals’ experimental conditions. This measurement was converted into a percentage and used as an index of fear (Fiorenza et al. 2012; Nachtigall et al. 2023, 2024).

### Cannulae placements

Cannulae placements were verified by infusing 1.0 µl of 4% (vol/vol) methylene blue into the cannulae after the last behavioral procedure, as described previously. Animals were then anesthetized and euthanized via decapitation 15 min later, and the spread of the dye was examined as an estimate of the drugs’ diffusion. Cannula placement was considered accurate if the spread was 1.0 mm3 from the intended infusion sites.

### Statistical analysis

Data are presented as mean ± standard error of the mean (S.E.M). The percentage of time spent freezing was analyzed using either the Student’s *t*-test or two-way ANOVA, with Treatment and Session as factors, to assess main effects and their potential interaction (Treatment X Session). Bonferroni’s post hoc test was applied to compare treatment groups within each session. Data normality was confirmed using the Shapiro-Wilk test, with *p* > 0.05 and homoscedasticity was verified using the Brown-Forsythe test, with *p* > 0.05. A p-value < 0.05 was considered statistically significant. All data were analyzed using GraphPad Prism^®^ software.

## Results

### Effects of the administration of the MAPK/ERK inhibitor U0126, in the CA1 region of the hippocampus on reconsolidation of contextual fear memory in male and female rats

We conducted our experiments using the contextual fear conditioning protocol, which was established as capable of promoting the reconsolidation of CFC memory (Nachtigall et al. 2023). To investigate the involvement of the MAPK/ERK pathway on the reconsolidation of CFC memory, male and female rats underwent a training session in the CFC paradigm, and 24 h later, they were presented with a reactivation session (3 min). Immediately after reactivation, the animals received intra-CA1 infusions of either Veh or U0126 (1.0 µl/side). Twenty-four hours later, memory was assessed in the retention test (3 min).

**Fig. 1:**
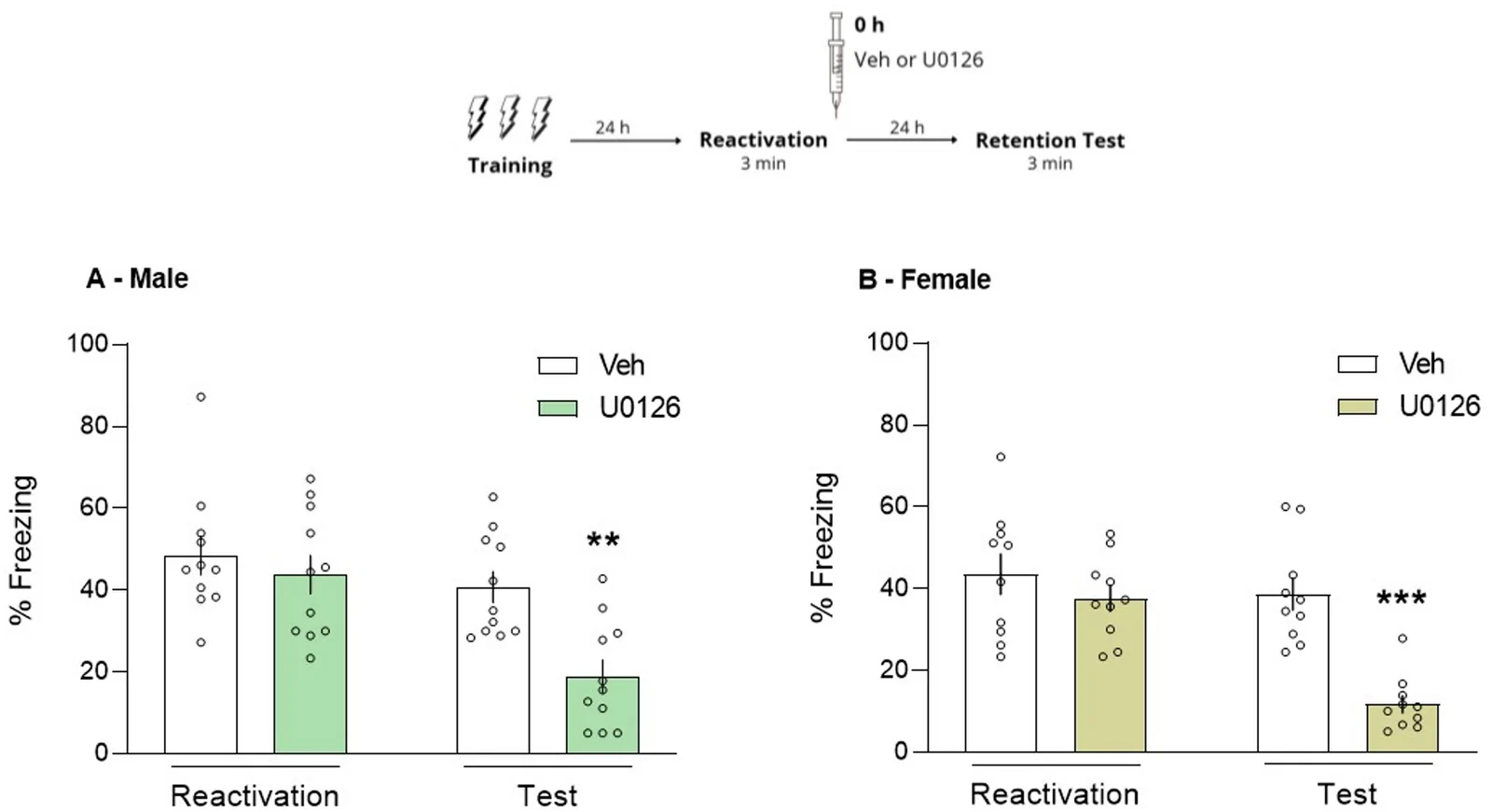
Intra-CA1 infusion of the MAPK/ERK pathway inhibitor, U0126, impairs the reconsolidation of CFC memory in both male and female rats. Fig 1A - Immediately after the CFC reactivation session, male rats received infusions (1.0 μl/side) of Veh (DMSO 1% in NaCl 0.9%) or the MAPK/ERK pathway inhibitor (U0126; 0.04 μg/side) in the CA1 region of the dorsal hippocampus. Twenty-four hours later, they underwent a 3-minute retention test. The animals that received U0126 exhibited lower freezing levels than the animals that received Veh. Fig 1B - Immediately after the CFC reactivation session, female rats received infusions (1.0 μl/side) of Veh (DMSO 1% in NaCl 0.9%) or the MAPK/ERK pathway inhibitor (U0126; 0.04 μg/side) in the CA1 region of the dorsal hippocampus. Twenty-four hours later, they underwent a 3-minute retention test. The females that received U0126 exhibited lower freezing levels than the animals that received Veh. The percentages of time spent freezing during the 3-minute reactivation session and the 3-minute retention test were analyzed by two-way repeated measures ANOVA followed by Bonferroni’s multiple comparison test. The data are presented as mean ± SEM. **p < 0.01 and ***p < 0.001 versus Veh on the retention test. n = 10-11 per group. (Upper) Schematic representation of the behavioral protocol used

As shown in Fig. 1A, male rats that received U0126 (0.04 µg/side) in the CA1 region of the hippocampus immediately after the reactivation session spent significantly less time freezing during the retention test when compared to the animals that received Veh, indicating long-term memory impairment. Two-way repeated-measures ANOVA demonstrated significant effects of Session (F(1,20) = 46.06, *p* < 0.001), of Treatment (F(1,20) = 5.61, *p* < 0.05) and Session × Treatment interaction (F(1,20) = 12.65, *p* < 0.01). Bonferroni’s multiple comparison test revealed that there was no difference between the groups during the reactivation session (*p* > 0.05), but there was a difference between the U0126 versus Veh group in the retention test (*p* < 0.01) (*n* = 11 per group).

Similarly, the female rats that received U0126 (0.04 µg/side) in the CA1 region of the hippocampus immediately after the reactivation session spent significantly less time freezing during the retention test compared to the female rats that received Veh (Fig. 1B), indicating long-term memory impairment. Two-way repeated-measures ANOVA demonstrated effects of Session (F(1,18) = 41.46, *p* < 0.001), of Treatment (F(1,18) = 12.29, *p* < 0.01) and Session × Treatment interaction (F(1,18) = 19.30 *p* < 0.001). Bonferroni’s multiple comparison test revealed that there was no difference between the groups during the reactivation session (*p* > 0.05), but there was a difference between the U0126 versus Veh group in the retention test (*p* < 0.001) (*n* = 10 per group).

These results suggest that the MAPK/ERK signaling pathway, in male and female rats, is involved in the process of contextual fear memory retention following U0126 infusion.

### Effect of U0126 administered in the CA1 region of the hippocampus 3 h after reactivation on reconsolidation of contextual fear memory in male and female rats

Considering that the process of memory reconsolidation can be modulated during a post-reactivation time window (Schmidt et al. 2017; Furini et al. 2020; Nachtigall et al. 2023), we conducted experiments evaluating the effect of U0126 infusion 3 h after the CFC memory reactivation session in male and female rats.

**Fig. 2:**
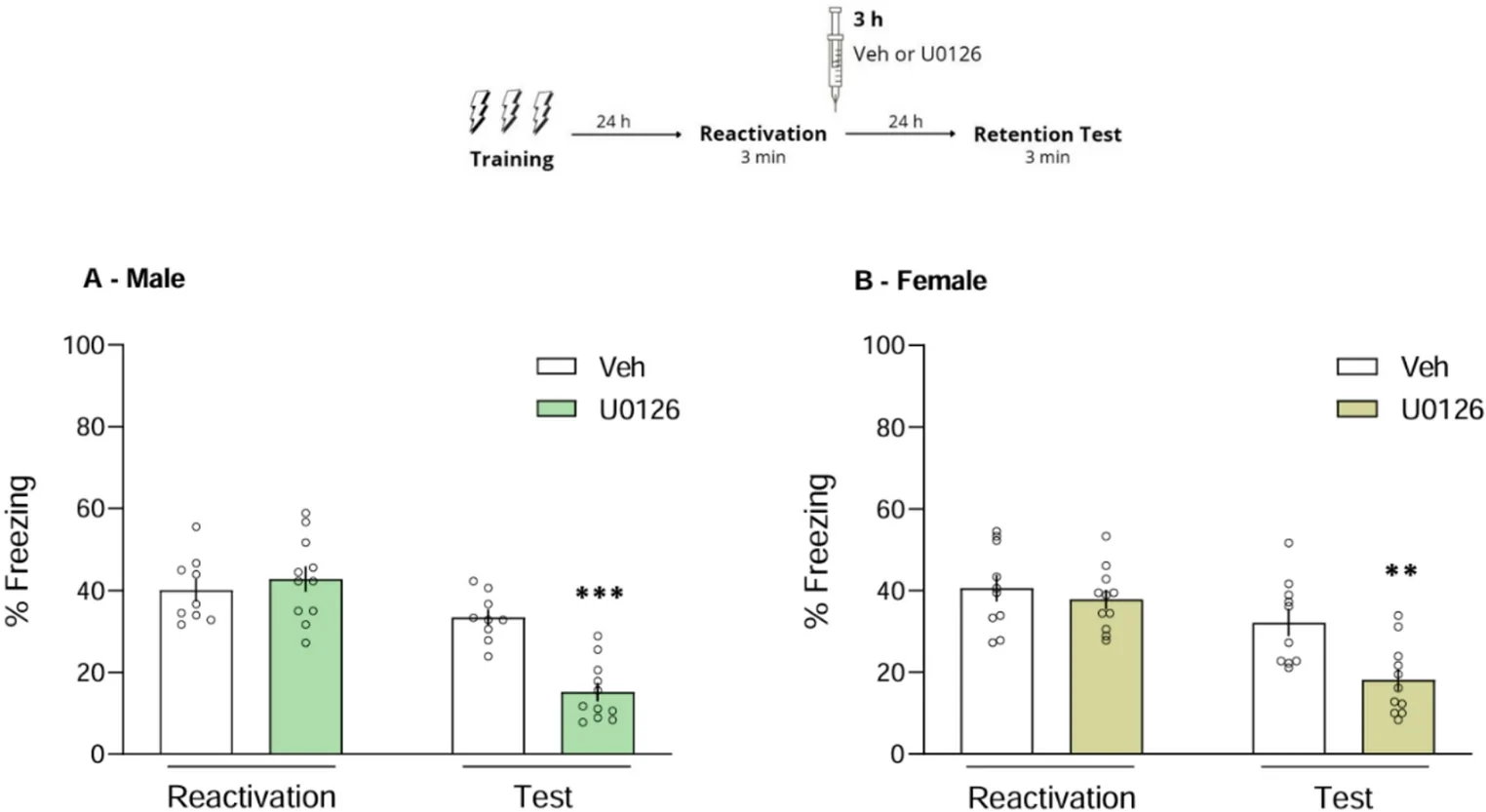
Intra-CA1 infusion of the MAPK/ERK pathway inhibitor, U0126, 3 h after reactivation impairs the reconsolidation of CFC memory in both male and female rats. Figure 2A - Three hours after the CFC reactivation session, male rats received infusions (1.0 µl/side) of Veh (DMSO 1% in NaCl 0.9%) or MAPK/ERK pathway inhibitor (U0126; 0.04 µg/side) in the CA1 region of the dorsal hippocampus. Twenty-four hours later, they underwent a 3-minute retention test. Animals that received U0126 exhibited lower freezing levels than male rats that received Veh. Figure 2B - Three hours after the CFC reactivation session, female rats received infusions (1.0 µl/side) of Veh (DMSO 1% in NaCl 0.9%) or MAPK/ERK pathway inhibitor (U0126; 0.04 µg/side) in the CA1 region of the dorsal hippocampus. Twenty-four hours later, they underwent a 3-minute retention test. Females that received U0126 exhibited lower freezing levels than animals that received Veh. The percentages of time spent freezing during the 3-minute reactivation session and the 3-minute retention test were analyzed by two-way repeated measures ANOVA followed by Bonferroni’s multiple comparison test. The data are presented as mean ± SEM. ***p < 0.001 and **p < 0.01 on retention test. *n* = 9–11 per group. (Upper) Schematic representation of the behavioral protocol used

As shown in Fig. 2A, male rats that received U0126 (0.04 µg/side) in the CA1 region of the hippocampus 3 h after the reactivation session spent significantly less time freezing during the retention test compared to the animals that received Veh, indicating long-term memory impairment. Two-way repeated-measures ANOVA demonstrated effects of Session (F(1,18) = 113.40, *p* < 0.001), of Treatment (F(1,18) = 5.69, *p* < 0.05) and Session × Treatment interaction (F(1,18) = 42.36, *p* < 0.001). Bonferroni’s multiple comparison test revealed that there was no difference between the groups during the reactivation session (*p* > 0.05), but there was a difference between the U0126 versus Veh group in the retention test (*p* < 0.001) (*n* Veh = 9 and *n* U0126 = 11).

Also, the female rats that received U0126 (0.04 µg/side) in the CA1 region of the hippocampus 3 h after reactivation session spent significantly less time freezing during the retention test compared to females that received Veh indicating long-term memory impairment (Fig. 2B). Two-way repeated-measures ANOVA demonstrated effects of Session (F(1,19) = 79.50, *p* < 0.001), of Treatment (F(1,19) = 5.03, *p* < 0.05) and Session × Treatment interaction (F(1,19) = 12.89, *p* < 0.01). Bonferroni’s multiple comparison test revealed that there was no difference between the groups during the reactivation session (*p* > 0.05), but there was a difference between the U0126 versus Veh group in the retention test (*p* < 0.01) (*n* Veh = 10 and *n* U0126 = 11).

These results suggest that MAPK/ERK signaling in the CA1 region of the hippocampus is required for the reconsolidation of CFC memory up to 3 h after the reactivation session in both male and female rats.

### Effect of U0126 administered in the CA1 region of the hippocampus 6 h after reactivation on reconsolidation of contextual fear memory in male and female rats

**Fig. 3:**
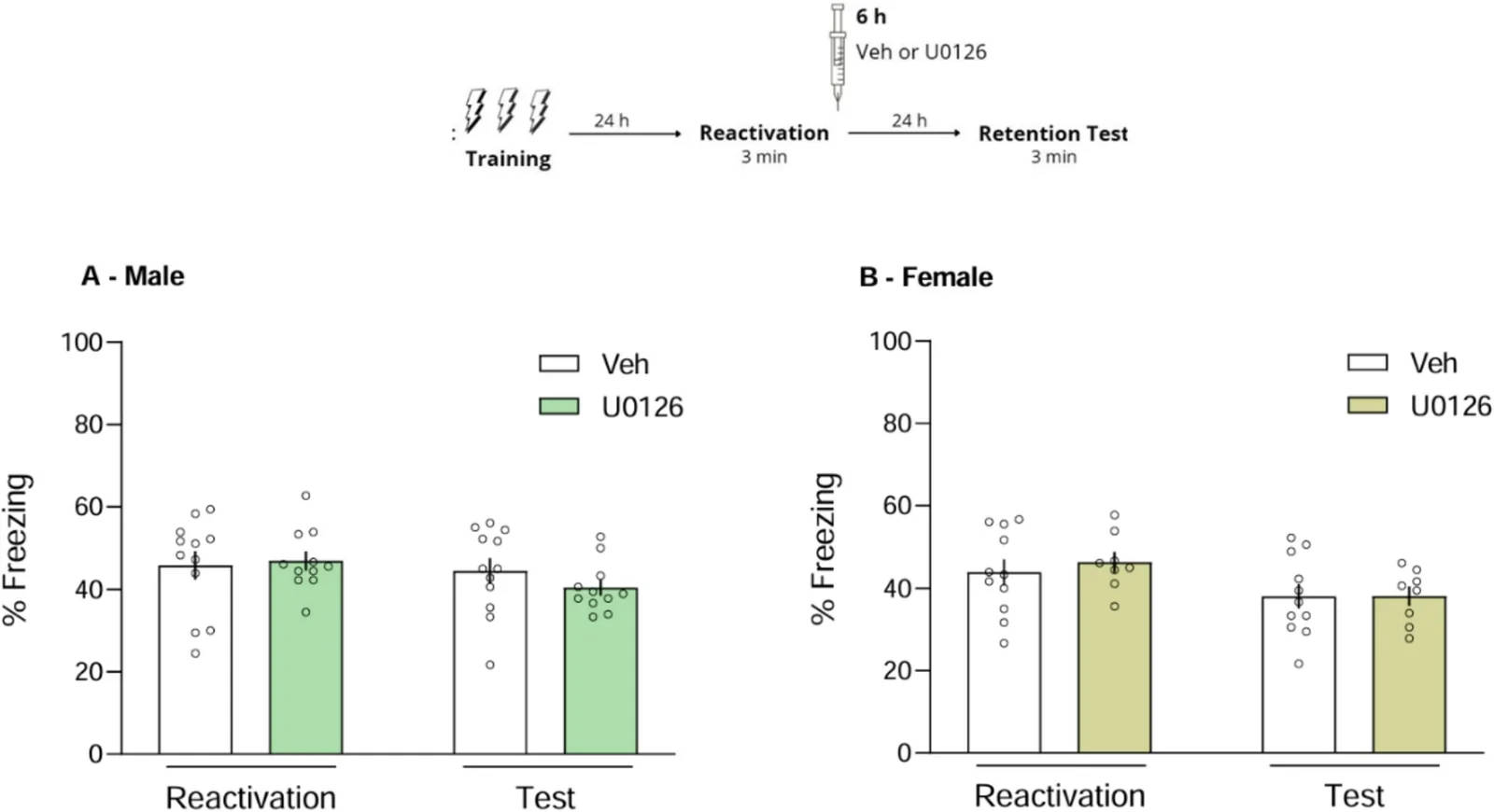
The intra-CA1 infusion of the MAPK/ERK pathway inhibitor, U0126, 6 h after reactivation, has no effect on reconsolidation of CFC memory in both male and female rats. Figure 3A - Six hours after the CFC reactivation session, male rats received infusions (1.0 µl/side) of Veh (DMSO 1% in NaCl 0.9%) or the MAPK/ERK pathway inhibitor (U0126; 0.04 µg/side) in the CA1 region of the dorsal hippocampus and were subjected to a 3-minute retention test 24 h later. Animals that received U0126 exhibited similar freezing levels compared to those that received Veh. Figure 3B - Six hours after the CFC reactivation session, female rats received infusions (1.0 µl/side) of Veh (NaCl 0.9%) or the MAPK/ERK pathway inhibitor (U0126; 0.04 µg/side) in the CA1 region of the dorsal hippocampus and were subjected to a 3-minute retention test 24 h later. Females that received U0126 exhibited similar freezing levels compared to those that received Veh. The percentages of time spent freezing during the 3-minute reactivation session and the 3-minute retention test were analyzed by two-way repeated-measures ANOVA. The data are presented as mean ± SEM. *n* = 8–12 per group. (Upper) Schematic representation of the behavioral protocol used

Considering the effect observed at 3 h after the reactivation session, we decided to test the effect of U0126 administration 6 h after the CFC memory reactivation session. As shown in Fig. 3A, male rats that received U0126 (0.04 µg/side) in the CA1 region of the hippocampus 6 h after the reactivation session exhibited similar levels of freezing during the retention test compared to the animals that received Veh, indicating that there was no effect on long-term memory. Two-way repeated-measures ANOVA demonstrated effect of Session (F(1,21) = 5.32, *p* = 0.03) with no effect of Treatment (F(1,21) = 0.18, *p* = 0.6758), and Session × Treatment interaction (F(1,21) = 2.24, *p* = 0.1493), (*n* Veh = 12 and *n* U0126 = 11).

As observed in male rats, female animals that received U0126 (0.04 µg/side) in the CA1 region of the hippocampus 6 h after reactivation also exhibited similar levels of freezing during the retention test compared to females that received Veh, indicating no effect on long-term memory (Fig. 3B). Two-way repeated-measures ANOVA demonstrated effect of Session (F(1,17) = 32.77, *p* < 0.001) with no effect of Treatment (F(1,17) = 0.10, *p* = 0.7521), and Session × Treatment interaction (F(1,17) = 0.99, *p* = 0.3318), (*n* Veh = 11 and *n* U0126 = 8).

These results suggest that MAPK/ERK signaling in the CA1 region of the hippocampus is not necessary for the reconsolidation of CFC memory 6 h after the reactivation session in both male and female rats.

### Effect of U0126 administered in the CA1 region of the hippocampus in the absence of the reactivation session in male and female rats

**Fig. 4:**
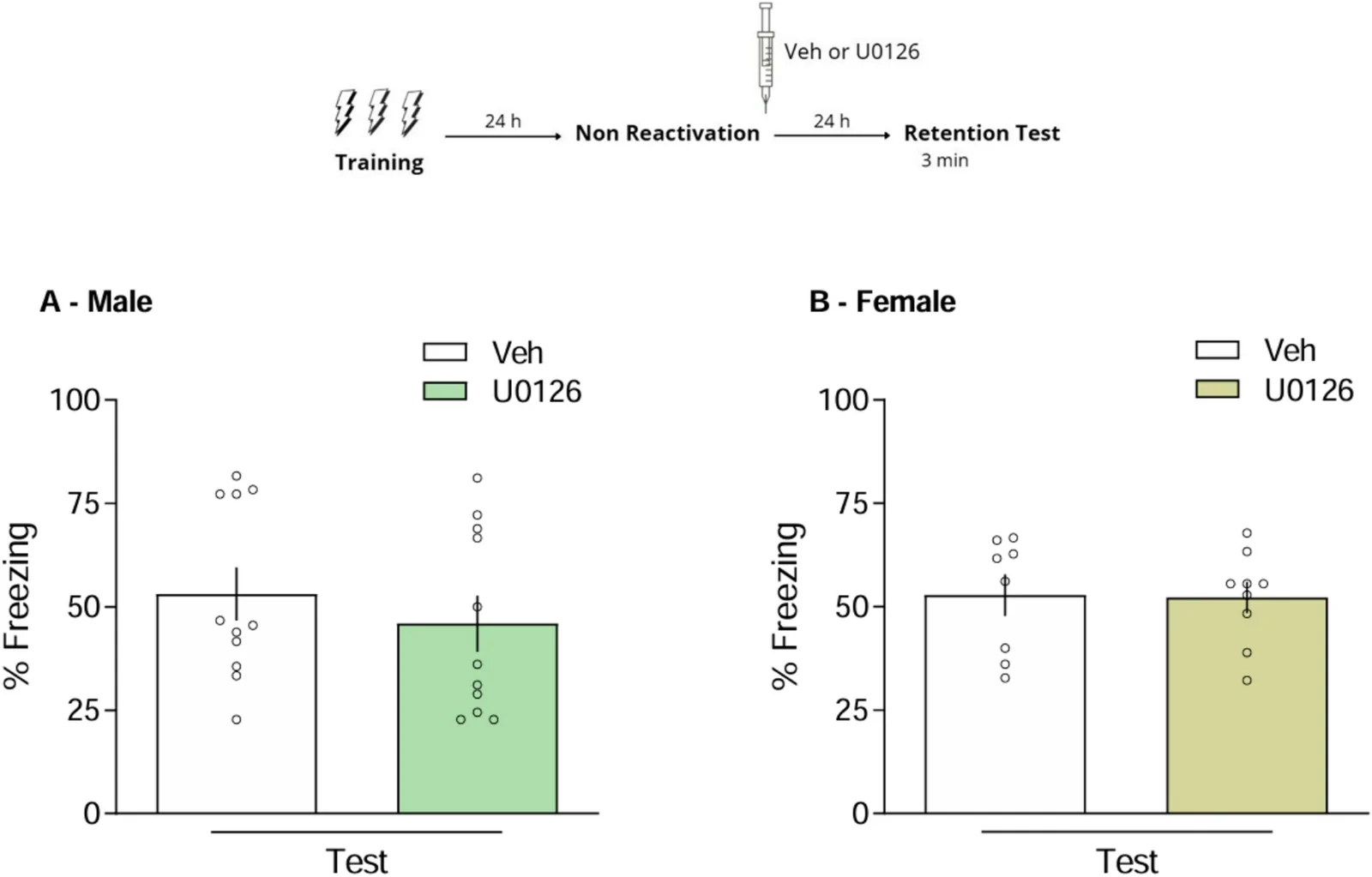
The intra-CA1 infusion of the MAPK/ERK pathway inhibitor, U0126, has no effect in the absence of a reactivation session in both male and female rats. Figure 4A - The MAPK/ERK pathway inhibitor, U0126, had no effect in the absence of retrieval when administered into the CA1 of male rats (*n* = 11). Figure 4B - The MAPK/ERK pathway inhibitor, U0126, had no effect in the absence of retrieval when administered into the CA1 of female rats (*n* = 8–9). Percentages of time spent freezing on the 3-min retention test were analyzed by Student’s *t*-test. Data are presented as mean ± SEM. (Upper) Schematic representation of the behavioral protocol used

Since U0126 was found to block memory reconsolidation, it was important to investigate whether it had an effect in the absence of a reactivation session, as it was possible that the observed impairment could be due to nonspecific effects. To investigate this, an infusion of U0126, 24 h after the training session was performed to evaluate the effect of U0126 without reactivation. The retention test was conducted on the following day. The absence of reactivation session prevented the impairing effect of U0126 in both male (Fig. 4A) and female rats (Fig. 4B). In Fig. 4A, Student’s *t*-test demonstrated that there was no difference between U0126 and Veh (t(20) = 0.7680, *p* = 0.45 on male rats, *n* = 11 per group) as well as Student’s *t*-test demonstrated that there was no difference between U0126 and Veh in female rats (t(15) = 0.0900, *p* = 0.9291; *n* Veh = 8, *n* U0126 = 9 per group) (Fig. 4B).

These results suggest that U0126-induced impairment of reconsolidation depends on memory destabilization following reactivation and that this effect is not due to nonspecific effects.

## Discussion

Here we demonstrated that the MAPK/ERK pathway is necessary for the reconsolidation of contextual fear memory in the CA1 region of the hippocampus up to 3 h following reactivation. Additionally, we observed no sex differences in this process, although potential sex-related variations cannot be completely excluded.

To obtain these results, we employed the contextual fear conditioning paradigm, a well-established experimental model that induces fear memory through aversive stimuli and allows its subsequent retrieval (Ledoux 2014). Fear is considered a physiological and behavioral response that is biologically adaptive to the real or anticipated presence of a threatening stimulus (Bishop 2007). In this regard, memory formed from an experience in a potentially dangerous environment helps reduce damage or ensures survival (Tovote et al. 2015). However, an organism’s reactivity based on fear memory can also trigger responses that influence behavior and contribute to the development of pathologies, such as Post-Traumatic Stress Disorder (PTSD), Obsessive-Compulsive Disorder (OCD), and various anxiety disorders, such as panic disorder and phobias (Kaminer et al. 2005; Sbardelloto et al. 2011; Kida 2022). Contextual fear conditioning is a model that allows the investigation of specific aspects of fear memory in animals by pairing a footshock (unconditioned stimulus that elicits fear) with the context of the training box in which the animal is placed (conditioned stimulus) (Todd et al. 2014).

The retention of information acquired from an experience undergoes the process of consolidation to become stable (Bolhuis 2000; Hamed et al. 2025). This process depends on the interaction between multiple neural circuits, which operate either independently or interconnected, involving neuronal plasticity, signaling cascades, and protein synthesis (Martin et al. 2000; Colombo 2004). In this context, studies show that the brain structures involved include the hippocampus, basolateral amygdala, and medial prefrontal cortex, which play crucial roles in the mechanics of memory, especially fear memory (Wang et al. 2005; Mucic et al. 2015; Furini et al. 2020). After consolidation, a memory can be retrieved through the presentation of cues, such as the CS. This reactivation can induce the destabilization of the initial memory, making it unstable again and susceptible to modifications and disruptions (Sara 2000; Alberini 2005; Furini et al. 2020; Hamed et al. 2025). Consequently, the engagement of cellular mechanisms and synaptic plasticity is required again to stabilize the destabilized memory. This process, known as reconsolidation, is particularly relevant for studies on fear memory (Dudai et al. 2015; Ma et al. 2023). Reconsolidation relies on cellular mechanisms like consolidation; however, it is a distinct process, aimed at maintaining, strengthening, or modifying memories (Berman and Dudai 2001; Taubenfeld et al. 2001; Dudai 2006).

Memory reconsolidation requires protein synthesis and gene expression (Kandel 2001). Research using protein synthesis inhibitors has demonstrated that reconsolidation can be blocked, showing that the reactivation of a memory creates a window of vulnerability during which it can be manipulated or weakened (Lima et al. 2009; Nachtigall et al. 2023). It is also demonstrated that reactivated or retrieved conditioned fear memories require protein synthesis mediated by CREB (Kida et al. 2002). CREB is a transcription factor responsible for the regulation of approximately 4,000 genes (Zhang et al. 2005). Thus, CREB can either activate or suppress gene transcription and induce or inhibit protein synthesis. CREB activation can occur through post-translational actions, binding of inhibitors, regulation by epigenetics, and phosphorylation. Several modulators capable of phosphorylating CREB are known, such as cytokines, lipids, growth factors, and calcium signaling. These extracellular modulators influence CREB through signaling transduction pathways, including the MAPK/ERK pathway (Steven et al. 2020).

Besnard et al. (2013) evaluated the importance of ERK1/2 activity during the retrieval of contextual fear memory and demonstrated that its activation is an early molecular event necessary for memory retrieval and subsequent reconsolidation. Notably, this activation occurs in the cytosol of hippocampal neurons, indicating that cytosolic ERK1/2 activity represents a critical early event mediating memory labilization and initiating reconsolidation. Evidence shows that cytosolic ERK1/2 activity is essential for memory reconsolidation, strengthening, and persistence after reactivation. In the hippocampus, ERK2 is activated in the cytosol 15 min post-reactivation and inhibited at 45 min. Pharmacological inhibition (PD098059) can either impair or enhance memory depending on the timing of application (Krawczyk et al. 2015). In the basolateral amygdala, ERK inhibition with U0126 immediately after reactivation reduces reinstatement and spontaneous recovery of heroin-associated memories, while later infusions have no effect (Li et al. 2022). In the dorsal hippocampus, ERK blockade 3 h after reactivation in the inhibitory avoidance task impaired long-term memory persistence without affecting immediate reconsolidation (Krawczyk et al. 2016). Also, inhibition of the MEK/ERK pathway prior to retrieval prevented the reconsolidation deficit observed in Zif268+/- mice, suggesting that blocking memory reactivation makes it resistant to reconsolidation (Besnard et al. 2013). In another study, Besnard et al. (2014) also revealed distinct ERK1/2 signaling dynamics between CFC memory consolidation and reconsolidation in different subregions of the hippocampus and amygdala. Notably, ERK1/2 phosphorylation in CA1 was higher after training (consolidation) than after retrieval (reconsolidation), while in the dentate gyrus (DG), activation patterns were similar. Additionally, they observed that the strength of an established CFC memory correlates with specific ERK1/2 activation patterns during retrieval (Besnard et al. 2014).

Furthermore, Zamorano and collaborators demonstrated that the retrieval of contextual fear memory reactivates ERK1/2 signaling in the same CA1 hippocampal neurons recruited during conditioning, highlighting the importance of the ERK1/2 pathway. Additionally, inhibition of ERK1/2 in CA1 shortly before context re-exposure blocked both memory retrieval and the reconsolidation initiated by retrieval, as anisomycin caused anterograde amnesia in the animals that received vehicle, but not ERK1/2 inhibitor (Zamorano et al. 2018).

Here, we blocked the MAPK/ERK pathway of the CA1 region at different time points after CFC memory reactivation and observed that this blockade had an effect when compared with the control group. Therefore, these results demonstrate that ERK1/2 can regulate both memory labilization and reconsolidation.

The freezing behavior observed in our study is unlikely to reflect nonspecific motor impairments or anxiety-like effects of the drug. Instead, it appears to involve specific mnemonic processes, as suggested by previous literature that has shown that U0126 alters memory traces through ERK/MAPK inhibition without producing changes in general locomotor activity or anxiety-like behavior (Einat et al. 2003; Tronson et al. 2008; Todorovic et al. 2009; Li et al. 2011; Maldonado et al. 2014; Zamorano et al. 2018; Fultz et al. 2021; de Carvalho et al. 2021). Collectively, these findings support the interpretation that the reduction in freezing observed with U0126 treatment reflects ERK pathway inhibition in memory processes rather than broad behavioral deficits.

Additionally, new guidelines for preclinical biological models have incorporated sex as a relevant biological variable (Clayton and Collins 2014; National Institutes of Health 2015). Preclinical studies evaluating the influence of sex on behavior have increasingly included female models, and in some cases, sex-specific differences have been observed (Zorzo et al. 2021; Huang et al. 2022; Dalferth et al. 2024).

A study conducted by Keiser and colleagues investigated sex differences in fear responses and the associated neural activity during memory retrieval in mice. The findings indicated that males exhibited increased c-Fos activity in the dorsal hippocampus, whereas females showed greater activation of the basal amygdala. Behaviorally, females demonstrated increased generalization of fear in similar contexts, while males exhibited more specific conditioned fear responses. This suggests a possible competition between hippocampus-and amygdala-dependent processes, modulated by sex. The hippocampus appears to play a role in reducing fear generalization, whereas the amygdala seems to be more involved in broad emotional responses and fear memory in females (Keiser et al. 2017).

Another study investigating the impact of sex on memory verified that male rats exhibit greater long-term retention of contextual fear memories compared with females, a difference that correlated with increased ERK phosphorylation in the ventral hippocampus in males 60 min post-conditioning. In contrast, females showed increased freezing responses to auditory cues, indicating a potential divergence in how each sex processes different fear memories. Interestingly, both sexes display ERK activation in the amygdala, suggesting that the hippocampus plays a more pronounced role in mediating these sex-specific differences (Mizuno et al. 2007; Gresack et al. 2009).

Notably, pharmacological inhibition of hippocampal calpain, a Ca2+-dependent cysteine protease involved in the Ca2 + signaling pathway, impaired long-term memory formation and disrupted reconsolidation of reactivated fear memories, in both male and female mice, suggesting that calpain is not involved in sex-specific molecular mechanisms underlying memory performance (Nagayoshi et al. 2017).

The studies mentioned above highlight the importance of considering sex in investigations of memory-related mechanisms. Considering this, we investigated whether an effect of the hippocampal MAPK/ERK signaling pathway on the reconsolidation of CFC memory would be observed in male and female rats. Although sex differences in memory have been widely studied, our work specifically examines MAPK/ERK signaling during the reconsolidation of contextual fear memory, a less explored phase. The CA1 region of the hippocampus was chosen due to its critical role in contextual memory, though future investigations comparing hippocampal effects with those in non-memory-related areas could further strengthen the understanding of the regional specificity of MAPK/ERK signaling in sex-dependent memory processes. Our findings indicate that the MAPK/ERK pathway in the CA1 region of the hippocampus plays a crucial role in the reconsolidation process, with a similar pattern observed in both sexes. Specifically, MAPK/ERK signaling is required to the reconsolidation of CFC memory up to 3 h after reactivation, but 6 h after reactivation, the pathway is no longer necessary in the studied region. Thus, under the present experimental conditions, no sex differences were observed regarding the requirement for MAPK/ERK signaling in reconsolidation. However, given the lack of estrous cycle monitoring and the absence of a power analysis for sex-by-treatment interactions, the possibility of sex-specific effects cannot be excluded. Furthermore, the reconsolidation impairment induced by U0126 depends on the destabilization of memory after reactivation, ruling out non-specific effects, and this pattern was consistent across both sexes. These findings suggest that, regardless of sex, MAPK/ERK signaling is critical during the early stages of reconsolidation but becomes dispensable at later time points. The results support that the effects of U0126 on reconsolidation are specifically related to memory destabilization rather than non-specific interference.

Some points warrant consideration. For example, the experimental control of sex-related variables: in our study, we did not align the experimental timeline with the estrous cycle of the females. Consequently, it was not possible to detect potential hormonal differences that might exist between the phases of the cycle, which could influence neurochemistry and responses to pharmacological interventions (Tanno et al. 2002; Keiser et al. 2017; Velasco et al. 2023). The absence of synchronization with the estrous cycle of females represents a methodological limitation, since hormonal fluctuations throughout the cycle can affect both memory processes and sensitivity to pharmacological interventions. Evidence from the literature shows that the effects of neurotransmitters and pharmacological agents on cognitive functions, such as working memory and fear memory formation, change according to the phase of the estrous cycle, particularly in relation to estrogen and progesterone peaks (Kirry et al. 2019; Do Nascimento et al. 2019; Berridge et al. 2022). For instance, Corticotropin-Releasing Factor (CRF) neurotransmission in the prefrontal cortex regulates working memory similarly in both sexes, but these effects are absent in females during proestrus, when ovarian steroid levels are at their maximum (Berridge et al. 2022). Moreover, the impact of prolonged stress on memory is more pronounced in females during the metestrus/diestrus phases, suggesting that the hormonal peak may confer protection against stress-induced deficits (Do Nascimento et al. 2019). Hormonal modulation also influences the response to muscarinic antagonists in the prefrontal cortex, with sex differences depending on the estrous cycle phase; for example, fear memory formation in females is only impaired by muscarinic antagonism during specific cycle phases (Kirry et al. 2019). Additionally, studies across different mouse strains have shown that avoidance behaviors and threat memory are influenced both by genetic background and by cyclical hormonal variations, reinforcing the need for strict control of the estrous cycle in experiments evaluating sex differences (Ryherd et al. 2023). Taken together, these findings indicate that the absence of estrous cycle control may mask sex-specific differences and yield results that do not accurately reflect the influence of ovarian hormones on neurochemistry and pharmacological responses. Future investigations should incorporate monitoring of the estrous cycle to enable more precise interpretations regarding sexually dimorphic mechanisms in cognitive and pharmacological processes.

Also, a compensatory effect may occur since males and females could employ different neurobiological strategies to achieve similar performance. Specifically, context is a critical factor contributing to sex differences in contextual fear conditioning, where males are reported to exhibit stronger fear conditioning (Mizuno et al. 2007; Gresack et al. 2009) and greater hippocampal activation during fear memory acquisition when compared to females (Kudo et al. 2004; Gresack et al. 2009). The influence of the experimental protocol on sex differences in fear memory may be more pronounced under higher stress conditions or in more challenging tasks. This is exemplified by the difference between sexes in response to painful stimuli (Araujo et al. 2018). Sex differences may not have emerged if the experimental protocol had not elicited a sufficiently stressful response. An additional consideration is that we relied on extensive previous literature to determine both the dose and the expected response of U0126 in blocking MEK activity (Schafe et al. 2000; Fischer et al. 2007; Tarpley et al. 2009; Kim et al. 2016; Chen et al. 2016; Wang et al. 2021), without performing a molecular validation of U0126 effects in CA1. Although previous studies consistently demonstrate that U0126 selectively inhibits MEK and reduces ERK1/2 phosphorylation, confirming pERK levels under our experimental conditions would have further strengthened the conclusions. Nevertheless, the behavioral data reported here provide convergent evidence with the well-established role of the MAPK/ERK pathway in memory reconsolidation. Additionally, it is important to note that our study was conducted in healthy animals, and we did not investigate the role of the MAPK/ERK pathway in contextual fear memory in the presence of pathological conditions. Therefore, the generalization of our findings on disease models should be done with caution. Previous studies suggest that the activation of MAPK/ERK pathways may differ between sexes in certain pathological contexts, such as stress-related disorders, neurodegenerative diseases, or chronic pain models (Barcelon et al. 2023; Schaffner et al. 2025). However, these aspects were beyond the scope of the present study and remain to be explored in future research. Nevertheless, the behavioral data reported here provide convergent evidence with the well-established role of the MAPK/ERK pathway in memory reconsolidation.

Thus, here we demonstrated that the MAPK/ERK signaling pathway is necessary for the reconsolidation of contextual fear memories in the hippocampus for up to 3 h after memory reactivation. Under the conditions tested, no sex differences were observed; nevertheless, sex-specific effects cannot be definitively ruled out. Additionally, our findings contribute to and expand the growing field of research on female subjects, which has increased in recent years, helping to bridge existing gaps in the understanding of memory reconsolidation mechanisms across sexes.

## Data Availability

The data supporting the study’s findings are available from the corresponding author upon reasonable request.

## References

[CR1] Alberini CM (2005) Mechanisms of memory stabilization: are consolidation and reconsolidation similar or distinct processes? Trends Neurosci 28:51–56. 10.1016/j.tins.2004.11.001 15626497

[CR2] Alberini CM (2011) The role of reconsolidation and the dynamic process of Long-Term memory formation and storage. 10.3389/fnbeh.2011.00012. Front Behav Neurosci 5:PMC3056265 21436877

[CR3] Araujo P, Coelho CA, Oliveira MG et al (2018) Neonatal sleep restriction increases nociceptive sensitivity in adolescent mice. Pain Physician 21:E137–E148 29565956

[CR4] Atkins CM, Selcher JC, Petraitis JJ et al (1998) The MAPK cascade is required for mammalian associative learning. Nat Neurosci 1:602–609. 10.1038/2836 10196568

[CR5] Barcelon E, Chung S, Lee J, Lee SJ (2023) Sexual dimorphism in the mechanism of pain central sensitization. 10.3390/cells12162028. Cells 12:2028 PMC10453375 37626838

[CR6] Berman DE, Dudai Y (2001) Memory extinction, learning anew, and learning the new: dissociations in the molecular machinery of learning in cortex. Science 291:2417–2419. 10.1126/science.1058165 11264539

[CR7] Berridge CW, Martin AJ, Hupalo S, Nicol SE (2022) Estrus cycle-dependent working memory effects of prefrontal cortex corticotropin-releasing factor neurotransmission. Neuropsychopharmacology 47:2016–2023. 10.1038/s41386-022-01349-7 PMC9556710 35618840

[CR8] Besnard A, Caboche J, Laroche S (2013) Recall and reconsolidation of contextual fear memory: differential control by ERK and Zif268 expression dosage. PLoS One 8:e72006. 10.1371/journal.pone.0072006 PMC3745394 23977192

[CR9] Besnard A, Laroche S, Caboche J (2014) Comparative dynamics of MAPK/ERK signalling components and immediate early genes in the hippocampus and amygdala following contextual fear conditioning and retrieval. Brain Struct Funct 219:415–430. 10.1007/s00429-013-0505-y 23389809

[CR10] Bishop SJ (2007) Neurocognitive mechanisms of anxiety: an integrative account. Trends Cogn Sci 11:307–316. 10.1016/j.tics.2007.05.008 17553730

[CR11] Blum S, Moore AN, Adams F, Dash PK (1999) A mitogen-activated protein kinase cascade in the CA1/CA2 subfield of the dorsal hippocampus is essential for long-term spatial memory. J Neurosci 19:3535–3544. 10.1523/JNEUROSCI.19-09-03535.1999 PMC6782236 10212313

[CR12] Bolhuis JJ (ed) (2000) Brain, perception, memory: advances in cognitive neuroscience. Oxford University Press, Oxford

[CR13] Bozon B, Kelly A, Josselyn SA et al (2003) MAPK, CREB and zif268 are all required for the consolidation of recognition memory. Philos Trans R Soc Lond B Biol Sci 358:805–814. 10.1098/rstb.2002.1224 PMC1693143 12740127

[CR14] Briscione MA, Jovanovic T, Norrholm SD (2014) Conditioned fear associated phenotypes as robust, translational indices of trauma-, stressor-, and anxiety-related behaviors. Front Psychiatry 5:88. 10.3389/fpsyt.2014.00088 PMC4104832 25101010

[CR15] Cestari V, Costanzi M, Castellano C, Rossi-Arnaud C (2006) A role for ERK2 in reconsolidation of fear memories in mice. Neurobiol Learn Mem 86:133–143. 10.1016/j.nlm.2006.01.003 16504549

[CR16] Chen T, Wang C, Sha S et al (2016) Simvastatin enhances spatial memory and long-term potentiation in hippocampal CA1 via upregulation of α7 nicotinic acetylcholine receptor. Mol Neurobiol 53:4060–4072. 10.1007/s12035-015-9344-6 26198568

[CR17] Clayton JA, Collins FS (2014) Policy: NIH to balance sex in cell and animal studies. Nature 509:282–283. 10.1038/509282a PMC5101948 24834516

[CR18] Clayton JA (2016) Studying both sexes: a guiding principle for biomedicine. FASEB J 30:519–524. 10.1096/fj.15-279554 PMC4714546 26514164

[CR19] Colombo PJ (2004) Learning-induced activation of transcription factors among multiple memory systems. Neurobiol Learn Mem 82:268–277. 10.1016/j.nlm.2004.07.005 15464409

[CR20] Dalferth TF, Nunes ML, Furini CRG (2024) Sleep deprivation in early life: cellular and behavioral impacts. Neurosci Biobehav Rev 159:105597. 10.1016/j.neubiorev.2024.105597 38387838

[CR21] Davis HP, Squire LR (1984) Protein synthesis and memory: a review. Psychol Bull 96:518–559. 10.1037/0033-2909.96.3.518 6096908

[CR22] de Carvalho CR, Lopes MW, Constantino LC et al (2021) The ERK phosphorylation levels in the amygdala predict anxiety symptoms in humans and MEK/ERK inhibition dissociates innate and learned defensive behaviors in rats. Mol Psychiatry 26:7257–7269. 10.1038/s41380-021-01203-0 34316004

[CR23] Debiec J, LeDoux JE, Nader K (2002) Cellular and systems reconsolidation in the hippocampus. Neuron 36:527–538. 10.1016/S0896-6273(02)01001-2 12408854

[CR24] Do Nascimento EB, Dierschnabel AL, De Macêdo Medeiros A et al (2019) Memory impairment induced by different types of prolonged stress is dependent on the phase of the estrous cycle in female rats. Horm Behav 115:104563. 10.1016/j.yhbeh.2019.104563 31377100

[CR25] Doyère V, Dębiec J, Monfils M-H et al (2007) Synapse-specific reconsolidation of distinct fear memories in the lateral amygdala. Nat Neurosci 10:414–416. 10.1038/nn1871 17351634

[CR26] Dudai Y, Eisenberg M (2004) Rites of passage of the engram: reconsolidation and the lingering consolidation hypothesis. Neuron 44:93–100. 10.1016/j.neuron.2004.09.003 15450162

[CR27] Dudai Y, Karni A, Born J (2015) The consolidation and transformation of memory. Neuron 88:20–32. 10.1016/j.neuron.2015.09.004 26447570

[CR28] Dudai Y (2004) The neurobiology of consolidations, or, how stable is the engram? Annu Rev Psychol 55:51–86. 10.1146/annurev.psych.55.090902.142050 14744210

[CR29] Dudai Y (2006) Reconsolidation: the advantage of being refocused. Curr Opin Neurobiol 16:174–178. 10.1016/j.conb.2006.03.010 16563730

[CR30] Duncia JV, Santella JB, Higley CA et al (1998) MEK inhibitors: the chemistry and biological activity of U0126, its analogs, and cyclization products. Bioorg Med Chem Lett 8:2839–2844. 10.1016/S0960-894X(98)00522-8 9873633

[CR31] Duvarci S, Nader K, LeDoux JE (2005) Activation of extracellular signal-regulated kinase- mitogen-activated protein kinase cascade in the amygdala is required for memory reconsolidation of auditory fear conditioning. Eur J Neurosci 21:283–289. 10.1111/j.1460-9568.2004.03824.x 15654867

[CR32] Eblen ST (2018) Extracellular regulated kinases: signaling from Ras to ERK substrates to control biological outcomes. Adv Cancer Res 138:99–142. 10.1016/bs.acr.2018.02.004 PMC6007982 29551131

[CR33] Einat H, Yuan P, Gould TD et al (2003) The role of the extracellular signal-regulated kinase signaling pathway in mood modulation. J Neurosci 23:7311–7316. 10.1523/JNEUROSCI.23-19-07311.2003 PMC6740453 12917364

[CR34] Fernandes HS, Popik B, De Oliveira Alvares L (2022) Effects of hippocampal IP3R inhibition on contextual fear memory consolidation, retrieval, reconsolidation and extinction. Neurobiol Learn Mem 188:107587. 10.1016/j.nlm.2022.107587 35051621

[CR35] Ferreira FF, Rodrigues FS, Schmidt SD et al (2019) Social support favors extinction and impairs acquisition of both short- and long-term contextual fear conditioning memory. Neurosci Lett 712:134505. 10.1016/j.neulet.2019.134505 31541725

[CR36] Fiorenza NG, Rosa J, Izquierdo I, Myskiw JC (2012) Modulation of the extinction of two different fear-motivated tasks in three distinct brain areas. Behav Brain Res 232:210–216. 10.1016/j.bbr.2012.04.015 22525015

[CR37] Fischer A, Radulovic M, Schrick C et al (2007) Hippocampal Mek/Erk signaling mediates extinction of contextual freezing behavior. Neurobiol Learn Mem 87:149–158. 10.1016/j.nlm.2006.08.003 PMC1839930 16979915

[CR38] Flint RW, Valentine S, Papandrea D (2007) Reconsolidation of a long-term spatial memory is impaired by cycloheximide when reactivated with a contextual latent learning trial in male and female rats. Neuroscience 148:833–844. 10.1016/j.neuroscience.2007.07.022 17766047

[CR39] Franzen JM, Vanz F, Werle I et al (2022) Cannabidiol impairs fear memory reconsolidation in female rats through dorsal hippocampus CB1 but not CB2 receptor interaction. Eur Neuropsychopharmacol 64:7–18. 10.1016/j.euroneuro.2022.08.002 36049316

[CR40] Fultz EK, Quadir SG, Martin D et al (2021) ERK-directed phosphorylation of mGlu5 gates Methamphetamine reward and reinforcement in Mouse. Int J Mol Sci 22:1473. 10.3390/ijms22031473 PMC7867251 33540617

[CR41] Furini CRG, Myskiw JC, Benetti F, Izquierdo I (2013) New frontiers in the study of memory mechanisms. Rev Bras Psiquiatr 35:173–177. 10.1590/1516-4446-2012-1046 23904024

[CR42] Furini CRG, Nachtigall EG, Behling JAK et al (2020) Molecular mechanisms in hippocampus involved on object recognition memory consolidation and reconsolidation. Neuroscience 435:112–123. 10.1016/j.neuroscience.2020.03.047 32272151

[CR43] Giachero M, Belén Sacson A, Belén Vitullo M et al (2024) Targeting fear memories: examining pharmacological disruption in a generalized fear framework. Neurobiol Learn Mem 213:107960. 10.1016/j.nlm.2024.107960 39004160

[CR44] Gresack JE, Schafe GE, Orr PT, Frick KM (2009) Sex differences in contextual fear conditioning are associated with differential ventral hippocampal extracellular signal-regulated kinase activation. Neuroscience 159:451–467. 10.1016/j.neuroscience.2009.01.009 19171181

[CR45] Hamed A, Kursa MB, Mrozek W et al (2025) Spatio-temporal mechanisms of consolidation, recall and reconsolidation in reward-related memory trace. Mol Psychiatry 30:1319–1328. 10.1038/s41380-024-02738-8 PMC11919705 39271752

[CR46] Huang B, Sun B, Yang R et al (2022) Long-lasting effects of postweaning sleep deprivation on cognitive function and social behaviors in adult mice. Neuropharmacology 215:109164. 10.1016/j.neuropharm.2022.109164 35716724

[CR47] Igaz LM, Vianna MRM, Medina JH, Izquierdo I (2002) Two time periods of hippocampal mRNA synthesis are required for memory consolidation of fear-motivated learning. J Neurosci 22:6781–6789. 10.1523/JNEUROSCI.22-15-06781.2002 PMC6758123 12151558

[CR48] Izquierdo I, Bevilaqua LR, Cammarota M (2006a) A arte de esquecer. estudos avançados 20:289–296

[CR49] Izquierdo I, Bevilaqua LRM, Rossato JI et al (2006b) Different molecular cascades in different sites of the brain control memory consolidation. Trends Neurosci 29:496–505. 10.1016/j.tins.2006.07.005 16872686

[CR50] Izquierdo I, Furini CRG, Myskiw JC (2016) Fear memory. Physiol Rev 96:695–750. 10.1152/physrev.00018.2015 26983799

[CR51] Izquierdo I, Medina JH (1993) Role of the amygdala, hippocampus and entorhinal cortex in memory consolidation and expression. Braz J Med Biol Res 26:573–589 7504967

[CR52] Jiang G, Hunter T (1999) Receptor signaling: when dimerization is not enough. Curr Biol 9:R568–R571. 10.1016/S0960-9822(99)80357-1 10469554

[CR53] Kaminer D, Seedat S, Stein DJ (2005) Post-traumatic stress disorder in children. World Psychiatry 4:121–125 PMC1414752 16633528

[CR54] Kandel ER (2001) The molecular biology of memory storage: a dialogue between genes and synapses. Science 294:1030–1038. 10.1126/science.1067020 11691980

[CR55] Keiser AA, Turnbull LM, Darian MA et al (2017) Sex differences in context fear generalization and recruitment of Hippocampus and Amygdala during retrieval. Neuropsychopharmacol 42:397–407. 10.1038/npp.2016.174 PMC5399239 27577601

[CR56] Kelly A, Laroche S, Davis S (2003) Activation of mitogen-activated protein kinase/extracellular signal-regulated kinase in hippocampal circuitry is required for consolidation and reconsolidation of recognition memory. J Neurosci 23:5354–5360. 10.1523/JNEUROSCI.23-12-05354.2003 PMC6741214 12832561

[CR57] Kida S, Josselyn SA, de Ortiz SP et al (2002) CREB required for the stability of new and reactivated fear memories. Nat Neurosci 5:348–355. 10.1038/nn819 11889468

[CR58] Kida S (2022) Mechanisms of fear memory processes. Brain Nerve 74:377–384. 10.11477/mf.1416202048 35437290

[CR59] Kim J, Szinte JS, Boulware MI, Frick KM (2016) 17β-estradiol and agonism of G-protein-Coupled Estrogen receptor enhance hippocampal memory via different Cell-Signaling mechanisms. J Neurosci 36:3309–3321. 10.1523/JNEUROSCI.0257-15.2016 PMC4792941 26985039

[CR60] Kirry AJ, Durigan DJ, Twining RC, Gilmartin MR (2019) Estrous cycle stage gates sex differences in prefrontal muscarinic control of fear memory formation. Neurobiol Learn Mem 161:26–36. 10.1016/j.nlm.2019.03.001 30851433

[CR61] Krawczyk MC, Blake MG, Baratti CM et al (2015) Memory reconsolidation of an inhibitory avoidance task in mice involves cytosolic ERK2 bidirectional modulation. Neuroscience 294:227–237. 10.1016/j.neuroscience.2015.03.019 25791227

[CR62] Krawczyk MC, Navarro N, Blake MG et al (2016) Reconsolidation-induced memory persistence: participation of late phase hippocampal ERK activation. Neurobiol Learn Mem 133:79–88. 10.1016/j.nlm.2016.06.013 27321160

[CR63] Kudo K, Qiao C-X, Kanba S, Arita J (2004) A selective increase in phosphorylation of Cyclic AMP response element-binding protein in hippocampal CA1 region of male, but not female, rats following contextual fear and passive avoidance conditioning. Brain Res 1024:233–243. 10.1016/j.brainres.2004.08.007 15451386

[CR64] Ledoux JE (2014) Coming to terms with fear. Proc Natl Acad Sci U S A 111:2871–2878. 10.1073/pnas.1400335111 PMC3939902 24501122

[CR65] Lee JLC, Everitt BJ, Thomas KL (2004) Independent cellular processes for hippocampal memory consolidation and reconsolidation. Science 304:839–843. 10.1126/science.1095760 15073322

[CR66] Lee JLC (2010) Memory reconsolidation mediates the updating of hippocampal memory content. Front Behav Neurosci 4:168. 10.3389/fnbeh.2010.00168 PMC2991235 21120142

[CR67] Li F, Wang X-S, Dai R-P et al (2011) The activation of NMDA receptor–ERK pathway in the central amygdala is required for the expression of morphine-conditioned place preference in the rat. Neurotox Res 20:362–371. 10.1007/s12640-011-9250-2 21681580

[CR68] Li H, Hu T, Zhang Y et al (2022) Extracellular signal-regulated kinase in the basolateral amygdala is required for reconsolidation of heroin-associated memory. Front Mol Neurosci 15. 10.3389/fnmol.2022.1020098 PMC9684340 36438183

[CR69] Lima RH, Rossato JI, Furini CR et al (2009) Infusion of protein synthesis inhibitors in the entorhinal cortex blocks consolidation but not reconsolidation of object recognition memory. Neurobiol Learn Mem 91:466–472. 10.1016/j.nlm.2008.12.009 19141326

[CR70] Ma H, Khaled HG, Wang X et al (2023) Excitation–transcription coupling, neuronal gene expression and synaptic plasticity. Nat Rev Neurosci 24:672–692. 10.1038/s41583-023-00742-5 PMC12024187 37773070

[CR71] Maeng LY, Milad MR (2015) Sex differences in anxiety disorders: interactions between fear, stress, and gonadal hormones. Horm Behav 76:106–117. 10.1016/j.yhbeh.2015.04.002 PMC4823998 25888456

[CR72] Maldonado NM, Espejo PJ, Martijena ID, Molina VA (2014) Activation of ERK2 in basolateral amygdala underlies the promoting influence of stress on fear memory and anxiety: influence of Midazolam pretreatment. Eur Neuropsychopharmacol 24:262–270. 10.1016/j.euroneuro.2013.10.005 24182621

[CR73] Maren S (2011) Seeking a spotless mind: extinction, deconsolidation, and erasure of fear memory. Neuron 70:830–845. 10.1016/j.neuron.2011.04.023 PMC3112357 21658578

[CR74] Marshall CJ (1995) Specificity of receptor tyrosine kinase signaling: transient versus sustained extracellular signal-regulated kinase activation. Cell 80:179–185. 10.1016/0092-8674(95)90401-8 7834738

[CR75] Martin SJ, Grimwood PD, Morris RGM (2000) Synaptic plasticity and memory: an evaluation of the hypothesis. Annu Rev Neurosci 23:649–711. 10.1146/annurev.neuro.23.1.649 10845078

[CR76] McGaugh JL (1966) Time-dependent processes in memory storage. Science 153:1351–1358. 10.1126/science.153.3742.1351 5917768

[CR77] McGaugh JL (2000) Memory–a century of consolidation. Science 287:248–251. 10.1126/science.287.5451.248 10634773

[CR78] Meyer F, Louilot A (2014) Consequences at adulthood of transient inactivation of the parahippocampal and prefrontal regions during early development: new insights from a Disconnection animal model for schizophrenia. Front Behav Neurosci 7. 10.3389/fnbeh.2013.00118 PMC3985036 24778609

[CR79] Mizuno K, Antunes-Martins A, Ris L et al (2007) Calcium/calmodulin kinase kinase β has a male-specific role in memory formation. Neuroscience 145:393–402. 10.1016/j.neuroscience.2006.11.056 17207577

[CR80] Mucic G, Sase S, Stork O et al (2015) Networks of protein kinases and phosphatases in the individual phases of contextual fear conditioning in the C57BL/6J mouse. Behav Brain Res 280:45–50. 10.1016/j.bbr.2014.11.024 25461266

[CR81] Nachtigall EG, De C. Myskiw J, Izquierdo I, Furini CRG (2024) Cellular mechanisms of contextual fear memory reconsolidation: role of hippocampal SFKs, TrkB receptors and GluN2B-containing NMDA receptors. Psychopharmacology 241:61–73. 10.1007/s00213-023-06463-y 37700085

[CR82] Nachtigall EG, de Freitas JDR, de C. Myskiw J, Furini CRG (2023) Role of hippocampal Wnt signaling pathways on contextual fear memory reconsolidation. Neuroscience 524:108–119. 10.1016/j.neuroscience.2023.05.028 37286160

[CR83] Nader K, Hardt O (2009) A single standard for memory: the case for reconsolidation. Nat Rev Neurosci 10:224–234. 10.1038/nrn2590 19229241

[CR84] Nader K, Schafe GE, Le Doux JE (2000) Fear memories require protein synthesis in the amygdala for reconsolidation after retrieval. Nature 406:722–726. 10.1038/35021052 10963596

[CR85] Nagayoshi T, Isoda K, Mamiya N, Kida S (2017) Hippocampal calpain is required for the consolidation and reconsolidation but not extinction of contextual fear memory. Mol Brain 10:61. 10.1186/s13041-017-0341-8 PMC5735908 29258546

[CR86] National Institutes of Health NI of H (NIH) (2015) Consideration of sex. as a Biological Variable in NIH-funded Research

[CR87] Ojea Ramos S, Feld M, Fustiñana MS (2022) Contributions of extracellular-signal regulated kinase 1/2 activity to the memory trace. Front Mol Neurosci 15:988790. 10.3389/fnmol.2022.988790 PMC9580372 36277495

[CR88] Orton RJ, Sturm OE, Vyshemirsky V et al (2005) Computational modelling of the receptor-tyrosine-kinase-activated MAPK pathway. Biochem J 392:249–261. 10.1042/BJ20050908 PMC1316260 16293107

[CR89] Paller KA, Voss JL (2004) Memory reactivation and consolidation during sleep. Learn Mem 11:664–670. 10.1101/lm.75704 PMC534694 15576883

[CR90] Paxinos G, Watson C (2014) Paxino’s and Watson’s The rat brain in stereotaxic coordinates, Seventh edition. Elsevier/AP, Academic Press is an imprint of Elsevier, Amsterdam ; Boston

[CR91] Raut SB, Joly F, Haass NK et al (2024) The clinically relevant MEK inhibitor Mirdametinib combined with D-cycloserine and prediction error disrupts fear memory in PTSD models. Transl Psychiatry 14:1–16. 10.1038/s41398-024-03190-6 PMC11655561 39695081

[CR92] Ryherd GL, Bunce AL, Edwards HA et al (2023) Sex differences in avoidance behavior and cued threat memory dynamics in mice: interactions between estrous cycle and genetic background. Horm Behav 156:105439. 10.1016/j.yhbeh.2023.105439 PMC10810684 37813043

[CR93] Sara SJ (2000) Retrieval and reconsolidation: toward a neurobiology of remembering. Learn Mem 7:73–84. 10.1101/lm.7.2.73 10753974

[CR94] Sbardelloto G, Schaefer LS, Justo AR, Haag Kristensen C (2011) Transtorno de estresse pós-traumático: evolução Dos critérios diagnósticos e prevalência. Psico-USF 16:67–73. 10.1590/S1413-82712011000100008

[CR95] Schafe GE, Atkins CM, Swank MW et al (2000) Activation of ERK/MAP kinase in the amygdala is required for memory consolidation of pavlovian fear conditioning. J Neurosci 20:8177–8187. 10.1523/JNEUROSCI.20-21-08177.2000 PMC6772720 11050141

[CR96] Schaffner SL, Tosefsky KN, Inskter AM et al (2025) Sex and gender differences in the molecular etiology of Parkinson’s disease: considerations for study design and data analysis. Biol Sex Differ 16:7. 10.1186/s13293-025-00692-w PMC11789417 39901234

[CR97] Schmidt SD, Furini CRG, Zinn CG et al (2017) Modulation of the consolidation and reconsolidation of fear memory by three different serotonin receptors in hippocampus. Neurobiol Learn Mem 142:48–54. 10.1016/j.nlm.2016.12.017 28034785

[CR98] Shaywitz AJ, Greenberg ME (1999) CREB: a stimulus-induced transcription factor activated by a diverse array of extracellular signals. Annu Rev Biochem 68:821–861. 10.1146/annurev.biochem.68.1.821 10872467

[CR99] Shimizu E, Tang Y-P, Rampon C, Tsien JZ (2000) NMDA receptor-dependent synaptic reinforcement as a crucial process for memory consolidation. Science 290:1170–1174. 10.1126/science.290.5494.1170 11073458

[CR100] Squire LR, Genzel L, Wixted JT, Morris RG (2015) Memory consolidation. Cold Spring Harb Perspect Biol 7:a021766. 10.1101/cshperspect.a021766 PMC4526749 26238360

[CR101] Steven A, Friedrich M, Jank P et al (2020) What turns CREB on? And off? And why does it matter? Cell Mol Life Sci 77:4049–4067. 10.1007/s00018-020-03525-8 PMC7532970 32347317

[CR102] Tanno AP, Bianchi FJ, Marcondes FK (2002) Influência do Ciclo estral sobre a sensibilidade Da Resposta cronotrópica à Norepinefrina Em Ratas submetidas a estresse Agudo. Rev Bras Cienc Farm 38:63–70. 10.1590/S1516-93322002000100006

[CR103] Tarpley JW, Shlifer IG, Birnbaum MS et al (2009) Bilateral phosphorylation of ERK in the lateral and centrolateral amygdala during unilateral storage of fear memories. Neuroscience 164:908–917. 10.1016/j.neuroscience.2009.08.071 PMC2783525 19735699

[CR104] Taubenfeld SM, Milekic MH, Monti B, Alberini CM (2001) The consolidation of new but not reactivated memory requires hippocampal C/EBPβ. Nat Neurosci 4:813–818. 10.1038/90520 11477427

[CR105] Todd TP, Vurbic D, Bouton ME (2014) Behavioral and neurobiological mechanisms of extinction in Pavlovian and instrumental learning. Neurobiol Learn Mem 108:52–64. 10.1016/j.nlm.2013.08.012 PMC3946264 23999219

[CR106] Todorovic C, Sherrin T, Pitts M et al (2009) Suppression of the MEK/ERK signaling pathway reverses depression-like behaviors of CRF2-deficient mice. Neuropsychopharmacol 34:1416–1426. 10.1038/npp.2008.178 PMC2680273 18843268

[CR107] Tovote P, Fadok JP, Lüthi A (2015) Neuronal circuits for fear and anxiety. Nat Rev Neurosci 16:317–331. 10.1038/nrn3945 25991441

[CR108] Tronson NC, Schrick C, Fischer A et al (2008) Regulatory mechanisms of fear extinction and depression-like behavior. Neuropsychopharmacology 33:1570–1583. 10.1038/sj.npp.1301550 PMC2562608 17712345

[CR109] Velasco ER, Florido A, Perez-Caballero L et al (2023) The Impacts of Sex Differences and Sex Hormones on Fear extinction. In: Milad MR, Norrholm SD (eds) Fear extinction. Springer International Publishing, Cham, 105–132 10.1007/7854_2023_426 37528309

[CR110] Wang D, Shi S, Ren T et al (2021) U0126 pretreatment inhibits cisplatin-induced apoptosis and autophagy in HEI-OC1 cells and cochlear hair cells. Toxicol Appl Pharmacol 415:115447. 10.1016/j.taap.2021.115447 33577918

[CR111] Wang S-H, Ostlund SB, Nader K, Balleine BW (2005) Consolidation and reconsolidation of incentive learning in the amygdala. J Neurosci 25:830–835. 10.1523/JNEUROSCI.4716-04.2005 PMC6725620 15673662

[CR112] Zamorano C, Fernández-Albert J, Storm DR et al (2018) Memory retrieval re-activates Erk1/2 signaling in the same set of CA1 neurons recruited during conditioning. Neuroscience 370:101–111. 10.1016/j.neuroscience.2017.03.034 28366664

[CR113] Zarich N, Oliva JL, Martínez N et al (2006) Grb2 is a negative modulator of the intrinsic Ras-GEF activity of hSos1. Mol Biol Cell 17:3591–3597. 10.1091/mbc.E05-12-1104 PMC1525251 16760435

[CR114] Zhang X, Odom DT, Koo S-H et al (2005) Genome-wide analysis of cAMP-response element binding protein occupancy, phosphorylation, and target gene activation in human tissues. Proc Natl Acad Sci U S A 102:4459–4464. 10.1073/pnas.0501076102 PMC555478 15753290

[CR115] Zorzo C, Arias JL, Méndez M (2021) Recovering spatial information through reactivation: brain oxidative metabolism involvement in males and females. Neuroscience 459:1–15. 10.1016/j.neuroscience.2021.02.002 33571597

